# The Dipeptide Monoester Prodrugs of Floxuridine and Gemcitabine—Feasibility of Orally Administrable Nucleoside Analogs

**DOI:** 10.3390/ph7020169

**Published:** 2014-01-27

**Authors:** Yasuhiro Tsume, Blanca Borras Bermejo, Gordon L. Amidon

**Affiliations:** 1 Department of Pharmaceutical Science, University of Michigan, Ann Arbor, MI 48109, USA; 2 Facultat De Medicina, Universitat de Lleida, Lleida, Catalunya 21007, Spain

**Keywords:** gemcitabine prodrug, floxuridine prodrug, mouse *in situ* perfusion, pancreatic tumor cells, dipeptide

## Abstract

Dipeptide monoester prodrugs of floxuridine and gemcitabine were synthesized. Their chemical stability in buffers, enzymatic stability in cell homogenates, permeability in mouse intestinal membrane along with drug concentration in mouse plasma, and anti-proliferative activity in cancer cells were determined and compared to their parent drugs. Floxuridine prodrug was more enzymatically stable than floxuridine and the degradation from prodrug to parent drug works as the rate-limiting step. On the other hand, gemcitabine prodrug was less enzymatically stable than gemcitabine. Those dipeptide monoester prodrugs exhibited 2.4- to 48.7-fold higher uptake than their parent drugs in Caco-2, Panc-1, and AsPC-1 cells. Floxuridine and gemcitabine prodrugs showed superior permeability in mouse jejunum to their parent drugs and exhibited the higher drug concentration in plasma after *in situ* mouse perfusion. Cell proliferation assays in ductal pancreatic cancer cells, AsPC-1 and Panc-1, indicated that dipeptide prodrugs of floxuridine and gemcitabine were more potent than their parent drugs. The enhanced potency of nucleoside analogs was attributed to their improved membrane permeability. The prodrug forms of 5′-l-phenylalanyl-l-tyrosyl-floxuridine and 5′-l-phenylalanyl-l-tyrosyl-gemcitabine appeared in mouse plasma after the permeation of intestinal membrane and the first-pass effect, suggesting their potential for the development of oral dosage form for anti-cancer agents.

## 1. Introduction

The anti-cancer agents 2′,2′-difluoro-2′-deoxyuridine (gemcitabine, Gemzar^®^) and 5-fluoro-2′-deoxyuridine (floxuridine, FdUR), two nucleoside analogs, have been used to treat pancreatic, non-small-cell lung, and colon cancers as the first-line therapy [1,2,3,4]. The adverse effects associated with those chemotherapeutics are still unresolved and the minimization of side-effects and the maximization of therapeutic efficacy for chemotherapeutic agents have been investigated. The modification of physicochemical properties has been examined to improve the therapeutic index. One of those strategies is a prodrug strategy. In this strategy, the focus on anti-viral and anti-cancer drugs has been developing their orally available dosage forms. Oral bioavailability and metabolic disposition of anti-viral drugs and anti-cancer drugs has been improved by designing for the transporter-targeted-delivery [4,5,6,7,8,9,10,11,12,13].

Amino acid monoester and dipeptide monoester prodrugs have been synthesized, characterized, and their potential to improve the oral bioavailability examined as a part of the development for oral drug delivery [7,11,12,13,14,15,16,17,18]. Reportedly, amino acid monoester prodrugs as well as dipeptide monoester prodrugs are substrates for intake transporters such as PEPT1, PEPT2, and ATB^0,+^, and the carrier-mediated mechanism with those transporters improves their oral bioavailability [19,20,21,22,23,24]. PEPT1 has broad substrate specificity and is expressed in the GI tract [25,26]. This transporter can transport dipeptides, tripeptides, amino acid monoester prodrugs and β-lactam antibiotics [11,27,28,29,30,31,32,33]. Yang and coworkers demonstrated that the importance of PEPT1 transporter for the oral absorption of valacyclovir in PEPT1 knockout mice [34]. The delivery of amino acid monoester and dipeptide monoester prodrugs might be enhanced in pancreatic ductal cancer cells, Panc-1 and AsPC-1s, due to the overexpression of oligopeptide transporters [11,12,35]. Moreover, dipeptide prodrugs might have more potential for the development of oral anti-cancer drugs because of their superior affinity to the PEPT1 transporter [9,11,21].

The anti-cancer nucleoside analogs such as 5-fluorouracil (5-FU), floxuridine, and gemcitabine have been investigated and their mechanistic action is well understood [36,37,38,39]. The main administration for anti-cancer nucleoside analogs to treat cancer is an intravenous route due to low and erratic oral bioavailability and stability issues [40,41]. Moreover, those analogs are converted to pyrimidine structure by metabolic enzymes such as thymidine phosphorylase in many tissues [11,41,42]. Thus, higher doses of those anti-cancer agents are required to assure clinical efficacy and have greater potential for toxicity. The improved chemical and enzymatic stabilities of anti-cancer drugs might lead to the reduction of adverse effect by lowering doses. Additionally, the prodrug strategy has been adopted to target the disease and tissue specific enzymes to minimize its adverse effects and/or to maximize its therapeutic effects [43,44,45,46].

In this report, l-phenylalanyl-l-tyrosine was adopted as a dipeptide promoiety for the anti-cancer prodrugs to assess the feasibility of orally administrative nucleoside analogs, floxuridine and gemcitabine, because of its potential for enzyme-specific prodrug activation [47]. As the part of developing orally administrative cancer agents, we describe the stability and permeability of dipeptide monoester prodrugs of gemcitabine and floxuridine, as well as their anti-proliferation activity in pancreatic cancer cells, AsPC-1 and Panc-1 cells. Uptake studies were conducted with Caco-2, Panc-1, and AsPC-1 cells with both the presence and the absence of 10 mM Gly-Pro and permeability studies were also performed with *in situ* mouse jejunal perfusion to determine the effective permeability (P_eff_). Furthermore, the feasibility of developing orally administrable chemotherapeutic agents was assessed by measuring the drug concentration and drug species in plasma after the perfusion study. The chemical and enzymatic stabilities of dipeptide prodrugs of floxuridine and gemcitabine along with their parent drugs were also evaluated at physiological pH and in Caco-2, Panc-1, and AsPC-1 cell homogenates. The successful development of oral administrative anti-cancer drugs would improve the quality of life and drastically reduce the insurance costs for cancer patients [48].

## 2. Experimental Section

### 2.1. Materials

Gemcitabine was extracted from the lyophilized powder (Gemzar^®^) supplied by Eli Lilly Pharmaceuticals (Indianapolis, IN, USA). Floxuridine was obtained from Lancaster (Windham, NH, USA). The *tert*-butyloxycarbonyl (Boc)-protected dipeptide, Boc-l-phenylalanyl-l-tyrosine, was obtained from Chem-Impex (Wood Dale, IL, USA). High-performance liquid chromatography (HPLC) grade acetonitrile was obtained from Fisher Scientific (St. Louis, MO, USA). *N,N*-dicyclohexylcarbodiimide (DCC), *N,N*-dimethylaminopyridine (DMAP), trifluoroacetic acid (TFA), and all other reagents and solvents were purchased from Aldrich Chemical Co. (Milwaukee, WI, USA). Cell culture reagents were obtained from Invitrogen (Carlsbad, CA, USA) and cell culture supplies were obtained from Corning (Corning, NY, USA) and Falcon (Lincoln Park, NJ, USA). All chemicals were either analytical or HPLC grade.

### 2.2. The Synthesis of Gemcitabine Prodrug and Floxuridine Prodrug

The synthesis and characterization of 5′-monoamino acid ester prodrugs of gemcitabine and floxuridine and 5′-dipeptide ester prodrugs of floxuridine have been reported previously [7,11,13]. Briefly, Boc-protected dipeptide, Boc-l-phenylalanyl-l-tyrosine, (1.1 mmol), DCC (1.1 mmol), and DMAP (0.1 mmol) were allowed to react with gemcitabine or floxuridine (1 mmol) in 7 mL of dry DMF for 24 h. The reaction progress was monitored by thin layer chromatography (TLC) (ethyl acetate). The reaction mixture was filtered and dichloromethane (DCM) was removed under vacuum at 40 °C. The residue was extracted with ethyl acetate (30 mL) and washed with water (2 × 20 mL), and saturated NaCl (20 mL). The organic layer was dried over MgSO_4_ and concentrated under vacuum. The reaction yielded a mixture of 3′-monoester, 5′-monoester, and 3′,5′-diester gemcitabine prodrugs and floxuridine prodrugs. The three spots observed on TLC were separated and purified using column chromatography (dichloromethane (DCM)/methanol, 20:1). Fractions from each spot were concentrated under vacuum separately. The Boc group was cleaved by treating the residues with 5 mL TFA/DCM (1:1). After 4 h, the solvent was removed and the residues were reconstituted with water and lyophilized. The TFA salts of dipeptide prodrugs of gemcitabine and floxuridine were obtained as white fluffy solids. The yields of 5′-dipeptide monoester gemcitabine prodrug and 5′-dipeptide monoester floxuridine prodrug were ~30% and ~35%, respectively. HPLC was used to evaluate the prodrug purity. Prodrugs were between 92%–99% pure. These prodrugs were easily separated from parent drug by HPLC. Electrospray ionization mass spectra (ESI-MS) were obtained on a Micromass LCT ESI-MS. The observed molecular weights of all prodrugs were found to be consistent with that required by their structure. The structural identity of the prodrugs was then confirmed using proton nuclear magnetic resonance (^1^H-NMR) spectra obtained on a 300 MHz Bruker DPX-300 NMR spectrometer. The structural identity of 5′-l-phenylalanyl-l-tyrosyl-floxuridine has been reported previously [11].

*5′-l-Phenylalanyl-l-tyrosyl-gemcitabine*. Yield, 30%; percent purity: 92%; ^1^H-NMR (DMSO-*d_6_*) δ, 11.40 (m, 1H), 9.27 (m, 2H), 8.95 (m, 1H), 8.36–8.06 (m, 4H), 7.48–6.97 (m, 7H), 6.79–6.56 (m, 2H), 6.17 (m, 1H), 4.74–4.60 (m, 2H), 4.45 (m, 1H), 4.11 (m, 2H), 3.91–3.56 (m, 2H), 3.28–2.78 (m, 3H), 2.01–1.97 (m, 2H); ESI-MS 574.4 (M+H)^+^.

### 2.3. Cell Culture

AsPC-1 cells (passages 30–40) from American Type Culture Collection (Rockville, MD, USA) were routinely maintained in RPMI-1640 containing 10% fetal bovine serum. Caco-2 cells (passages 33–38) and Panc-1 (passages 20–35) from American Type Culture Collection) were routinely maintained in DMEM containing 10% fetal bovine serum. Cells were grown at 37 °C at 5% CO_2_ and 90% relative humidity in antibiotic-free media to avoid the possible transport interference by antibiotics.

### 2.4. Hydrolysis Studies

#### 2.4.1. Enzymatic Stability

Confluent Caco-2, Panc-1, and AsPC-1cells were rinsed twice with phosphate buffered saline (PBS). The cells were lysed with phosphate buffer (pH 7.4) by ultrasonication (Micro ultrasonic cell disrupter Model KT40, Kontes, Vineland, NJ, USA), and pelleted by centrifugation for 5 min at 1,000 × *g*. Protein amount was quantified with the Bio-Rad (Hercules, CA, USA) DC Protein Assay using bovine serum albumin as a standard. The amount of protein was adjusted to 500 µg/mL and hydrolysis reactions were carried out in 96-well plates (Corning). Caco-2, Panc-1, and AsPC-1 cell suspensions (250 µL) were placed in triplicate wells, the reactions were started with the addition of substrate, and cells were incubated at 37 °C for 120 min. At the desired time point, sample aliquots (35 µL) were removed and added to acetonitrile (ACN, 150 µL) with 0.1% TFA. The mixtures were filtered with a 0.45 µm filters at 1,000 × *g* for 10 min at 4 °C. The filtrate was then analyzed via reverse-phase HPLC.

#### 2.4.2. Chemical Stability

The nonenzymatic hydrolysis of the prodrugs was determined as described above, except that each well contained pH 7.4 phosphate buffers (10 mmol/L) instead of cell homogenate.

### 2.5. Uptake Studies

Caco-2, Panc-1, and AsPC-1 cells were grown on a 6-well plate for 18, 6, and 6 days, respectively. Wells were rinsed with MES (pH 6.0) buffer twice. Fresh MES buffer was reapplied to each well and incubated at 37 °C for 15 min. Each drug was individually tested from freshly prepared solutions in MES buffer (0.1 mM, total 0.3 mL) with the presence and the absence of 10 mM glycyl-proline (Gly-Pro). The solution was placed in each well and incubated at 37 °C for 60 min. Drug solution was removed and 3 mL of ice-cold PBS was immediately placed in each well. Each well was rinsed with 3 mL of cold-PBS twice and 0.5 mL of methanol/H_2_O (1:1) containing 0.1% TFA was placed in each well. The cell suspension was collected and transferred to a new tube. Those tubes were spun at 1,000 × *g* at 4 °C for 5 min. The supernatant was mixed with equal amount of water with either 0.1% formic acid or 0.1% ammonium hydroxide for LC-MS analysis. The cell pullets were used to determine protein amount with the Bio-Rad DC Protein Assay using bovine serum albumin as a standard.

### 2.6. Solution for Single-Pass Intestinal Perfusion

The perfusion buffer (pH6.0) consisted of 145 mM NaCl, 0.5 mM MgCl_2_, 1 NaH_2_PO_4_, 1 mM CaCl_2_, 3 mM KCl, 5 mM glucose, and 5 mM MES. The pH of the buffer was adjusted to pH6.0. This perfusion buffer also contained phenol-red (14 µM) as a non-absorbable marker for water flux measurements.

### 2.7. Single-Pass Intestinal Perfusion Studies in Mice

All animal experiments were conducted using protocols approved by the University of Michigan Committee of Use and Care of Animals (UCUCA). Female BALB/c mice (Charles River, IN, USA) weighing 20–25 g were used for all perfusion studies. Prior to each experiment, mice were fasted overnight with free access to water.

The procedure for the *in situ* single-pass intestinal perfusion is previously reported [49]. Briefly, mice were anesthetized with an i.m. injection of ketamine-xylazine mixture (ketamine: 80–120 mg/kg, xylazine: 5–10 mg/kg) and placed on a heated pad maintained at 37 °C. The abdomen was opened by a midline incision and a jejunal segment (approximately 10 cm) was carefully exposed to cannulate both ends with flexible PVC tubing (2.06 mm i.d., Fisher Scientific Inc., Pittsburgh, PA, USA). All solutions were incubated in a 37 °C water-bath. The isolated segment was rinsed with blank perfusion buffer to clean out any residual debris.

At the start of the study, the test compound (100 µM) in perfusion buffer (pH 6.0) including phenol red was perfused through the intestinal segment (Watson-Marlow Pump 323S, Watson-Marlow Bredel Inc., Wilimington, MA, USA), at the flow rate of 0.1 mL/min. The perfusion buffer was perfused for 30 min to assure steady-state and samples were taken in every 10 min for 90 min. At the end of perfusion, blood samples were collected by cardiac puncture. Blood samples with heparin were centrifuged at 1,000 × *g* for 10 min at 4 °C to collect plasma samples for LC-MS analysis. Following the termination of the experiment, the length of each perfused intestinal segment was measured.

### 2.8. Cell Proliferation Assays

Cell proliferation studies were conducted with Panc-1 and AsPC-1 cells. The cells were seeded into 96-well plates at 80,000 cells per well and allowed to attach/grow for 48 h before drug solutions were added. The culture medium (DMEM +10% fetal bovine serum or RPMI-1640 +10% fetal bovine serum) was removed and the cells were gently washed once with sterile pH 6.0 uptake buffer. Floxuridine, 5′-l-phenylalanyl-l-tyrosyl-floxuridine, gemcitabine and 5′-l-phenylalanyl-l-tyrosyl-gemcitabine were 2-fold serially diluted in pH 6.0 uptake buffer from 5 to 0.078 mmol/L. Buffer alone was used as a 100% viability control. The wash buffer was removed and 30 µL drug solution per well was added and incubated at 37 °C for 2 h in the cell incubator. After this time, the drug solutions were removed and the cells were again gently washed twice with sterile uptake buffer. The culture medium was then added to each well after washing. The cells were allowed to recover for 24 h before evaluating cell viability via 2,3-bis[2-methoxy-4-nitro-5-sulfophenyl]-2H-tetrazolium-5-carboxanilide inner salt (XTT) assays. A mixture (30 µL) containing XTT (1 mg/mL) in sterile RPMI-1640 without phenol red and phenazine methosulfate (N-methyldibenzopyrazine methyl sulfate in sterile PBS, 0.383 mg/mL) reagents were added to the cells and incubated at 37 °C for 1 h, after which the absorbance at 450 nm was read. The concentrations required to inhibit cell growth by 50% (GI_50_) were calculated using GraphPad Prism version 3.0 by nonlinear data fitting.

### 2.9. Data Analysis

The net water flux in the mouse perfusion studies was determined using phenol red (14 µM), a non-absorbed and non-metabolized marker. The measured C_out_/C_in_ ratio of test compound was corrected for water flux according to the following equation:

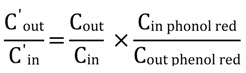

where C_in phenol red_ and C_out phenol red_ are equal to the concentration of phenol red in the inlet and the outlet samples, respectively. The effective permeability (P*_eff_*; cm/s) through the mouse intestinal wall in the single-pass intestinal perfusion studies was determined according to the following equation:

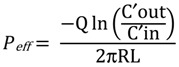

where Q is the perfusion buffer flow rate (0.1 mL/min), C′_out_/C′_in_ is the ratio of the outlet/inlet concentration of test compound that is adjusted for water transport, R is the radius of the intestinal segment (set to 0.1 cm), and L is the length of the perfused intestinal segment. 

### 2.10. HPLC Analysis

The stability samples of prodrugs and their metabolites, and perfusion samples were determined on an Agilent HPLC system (Agilent Technologies, Santa Clara, CA, USA). The HPLC system consisted of Agilent pumps (1100 series), an Agilent autosampler (1200 series), and an Agilent UV-Vis detector (1100 series) controlled by Chemstation^®^ 32 software (version B.01.03). Samples were resolved in an Agilent Eclipse Plus C18 reverse-phase column (3.5 µm, 4.6 × 75 mm) equipped with a guard column. The mobile phase consisted of 0.1% TFA/water (Solvent A) and 0.1% TFA/acetonitrile (Solvent B) with the solvent B gradient changing from 0%–56% at a rate of 2%/min during a 15 min run for gemcitabine and gemcitabine prodrugs. Standard curves generated for each prodrug and their parent drug were utilized for quantitation of integrated area under peaks. The detection wavelength was 254 nm and spectra were acquired in the 220–380 nm wavelength range. The detection wavelength for phenol red was 430 nm.

### 2.11. LC-MS Analysis

The LC-MS analytical method of 5′-mono amino acid ester prodrugs of floxuridine has been reported previously [50]. The LC-MS analysis of dipeptide monoester prodrugs of floxuridine and gemcitabine along with their parent drugs was modified and performed in a similar manner. Briefly, LC-MS analysis of the uptake drug amount was performed in triplicate on LCMS-2010EV (Shimadzu Scientific Instruments, Kyoto, Japan) equipped with an ESI (electrospray ionization) source. The Shimadzu LC-MS system consisting of Shimadzu LC-20AD pumps with DGU-20A in-line vacuum degasser units, and SIL-20A HT autosampler with an InertSustain C-18, 2.1 × 50 mm, 3 µm particle size, (GL Sciences, Torrance, CA, USA) was used for the separation and the effluent from the column was introduced directly to the ionization source. The system was controlled by Shimadzu LCMS solution software (version 3) to collect and process data. All samples were run with Solvent A and Solvent B with Solvent B gradient changing from 0%–90% at a rate of 13.8%/min over a 22 min run. The mobile phase consisted of 0.1% TFA/water (Solvent A) and 0.1% TFA/acetonitrile (Solvent B) for gemcitabine, gemcitabine prodrug and floxuridine prodrug. The mobile phase consisted of 0.1% ammonium hydroxide/water (Solvent A) and 0.1% ammonium hydroxide /acetonitrile (Solvent B) for floxuridine. The ESI probe was operated with a detector voltage of 1.5 kV, CDL temperature of 250 °C, heat block of 200 °C, and nebulizing gas flow of 1.2 mL/min in positive mode for gemcitabine prodrugs and their metabolites. The drying gas was N2 delivered at 0.1 MPa.

### 2.12. Statistical Analysis

Statistical analysis was performed by Student’s *t*-test for two groups. All results were expressed as the mean ± standard deviation (SD). A probability (*p*) of less than 0.05 is considered statistically significant.

## 3. Results and Discussion

### 3.1. Floxuridine and Gemcitabine Prodrugs

**Figure 1:**
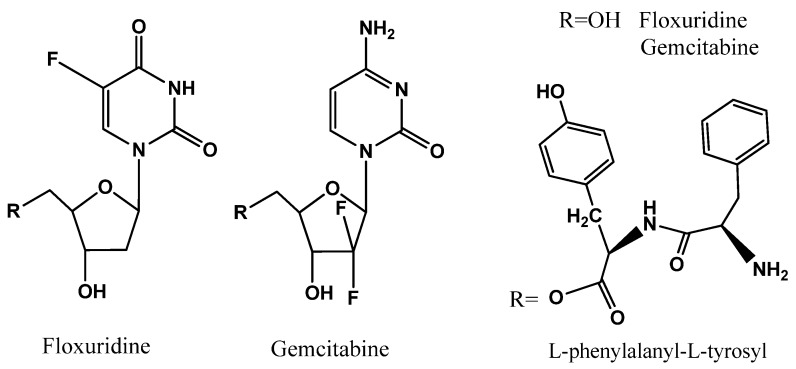
Dipeptide monoester prodrugs of floxuridine and gemcitabine.

The syntheses of the prodrugs and their characterization have been described in the previous report [7,11,12,13]. The structures and analytical data of those prodrugs were shown in Figure 1 and Table 1.

**Table 1:** Analytical data for dipeptide ester prodrugs of floxuridine and gemcitabine.

Prodrug	Purity (%) (HPLC)	ESI-MS (M+H)^+^		LogP ^a^
		Required	Observed	
Gemcitabine	95.3	263.2	263.9	−1.14
5′-L-Phenylalanyl-L-tyrosylgemcitabine	95.5 ^b^	574.5	574.4	1.04
Floxuridine	100.0	246.2	245.0	−0.51
5′-L-Phenylalanyl-L-tyrosyl-floxuridine	99.0	557.2	557.2	0.12

^a^ Calculated using BioLoom; ^b^ Mixture of 5′-l-phenylalanyl-l-tyrosylgemcitabine and its diastereomer.

### 3.2. The Stability of Floxuridine, 5′-l-Phenylalanyl-l-Tyrosyl-floxuridine, Gemcitabine, and 5′-l-Phenylalanyl-l-Tyrosyl-gemcitabine in Three Buffers (Acidic pH, SIF (pH 6.0), and pH 7.4), and Caco-2, Panc-1, and AsPC-1 Cell Homogenates

The experiments concerning drug and their prodrug stability were performed at 37 °C in 0.01 N HCl, SIF (pH 6.0), and pH 7.4 phosphate buffer. The estimated half-lives (t_1/2_) obtained from linear regression of pseudo-first-order plots of prodrug concentration *vs.* time for floxuridine, 5′-l-phenylalanyl-l-tyrosyl-floxuridine, gemcitabine, and 5′-l-phenylalanyl-l-tyrosyl-gemcitabine in 0.01 N HCl alone, SIF (pH 6.0), pH 7.4 phosphate buffer alone, and in Caco-2, Panc-1, and AsPC-1 cell homogenates are listed in Table 2. Prodrug metabolites such as floxuridine, 5-fluorouracil (5-FU), gemcitabine, 2′-deoxy-2′,2′-difluorouridine, uracil, and cytosine were monitored along with prodrug disappearance in this experiment. However, the mass balance could not be established because 5-FU, cytosine and uracil were metabolized even further and those metabolites could not be quantified by HPLC [11,50]. The specific kinetic parameters in drug/prodrug metabolism could not be determined without the mass balance and the data of enzymatic activities and their expression levels because those metabolic activities were simultaneously taken place. All tested drugs including dipeptide prodrugs showed the good chemical stability except 5′-l-phenylalanyl-l-tyrosyl-gemcitabine in 0.01N HCl, SIF, and pH 7.4 phosphate buffer. Gemcitabine was more chemically and enzymatically stable than another nucleoside analog, floxuridine (Table 2). 5′-l-Phenylalanyl-l-tyrosyl-floxuridine exhibited the best stability among tested compounds even though its parent drug, floxuridine, showed less stability compared to the other parent drug, gemcitabine. Gemcitabine prodrug exhibited 3.9- to 7.1-fold shorter half-lives in cell homogenates than its parent drug, gemcitabine, while floxuridine prodrug exhibited 7.3- to 9.3- fold longer half-lives in cell homogenates than its parent drug, floxuridine. The stability of 5′-l-phenylalanyl-l-tyrosyl-floxuridine was 1.3- and 7.4-fold better enzymatically in cell homogenates than one of 5′-l-phenylalanyl-l-tyrosyl-gemcitabine, while the stability of its parent drug, floxuridine, was 2.9- and 7.3-fold less enzymatically in cell homogenates than one of gemcitabine. The stability profiles of 5′-l-phenylalanyl-l-tyrosyl-gemcitabine in cell homogenates, Caco-2 cells, surrogate for intestine, Panc-1 and AsPC-1, surrogate for tumors, suggest that bioconversion of 5′-l-phenylalanyl-l-tyrosyl-gemcitabine to the parent drug would be much faster than metabolism of its parent drug, gemcitabine. The enzymatic stability of 5′-l-phenylalanyl-l-tyrosyl-floxuridine was significantly enhanced compared to one of floxuridine suggesting that the 5′ position of dipeptide has the catalytic role for its metabolic cascade for floxuridine and the cleavage of the ester bond is the rate-limiting step in the metabolic pathway of floxuridine prodrug.

**Table 2:** Stability of floxuridine, floxuridine prodrug, gemcitabine and gemcitabine prodrugs in 0.01N HCl, SIF (pH 6.0), pH 7.4 Buffer and biological media (mean ± SD, *n* = 3).

Prodrug	0.01 N HCl t_1/2_ (min)	SIF pH 6.0 t_1/2_ (min)	Buffer pH 7.4 t_1/2_ (min)	Caco-2 cell homogenates t_1/2_ (min)	Panc-1 cell homogenates t_1/2_ (min)	AsPC-1 cell homogenates t_1/2_ (min)
Gemcitabine	>120	>120	>120	105.0 ± 6.1	>120	33.7 ± 14.5
5′-l-Phenylalanyl-l-tyrosyl-gemcitabine	>120	>120	33.6 ± 1.4	14.7 ± 4.4	30.2 ± 1.1	8.1 ± 0.6
Floxuridine	>120	>120	>120	14.3 ± 7.0 ^a^	41.7 ± 6.8	6.4 ± 3.2 ^a^
5′-l-Phenylalanyl-l-tyrosyl-floxuridine	>120	>120	>120	103.8 ± 55.5 ^b^	40.4 ± 0.2	59.7 ± 1.4 ^b^

^a^ Reference [51]; ^b^ Reference [11].

### 3.3. Uptake Study of Floxuridine, 5′-l-Phenylalanyl-l-Tyrosyl-floxuridine, Gemcitabine, and 5′-l-Phenylalanyl-l-Tyrosyl-gemcitabine in Caco-2, Panc-1, and AsPC-1 Cells with Both the Presence and the Absence of 10 mM Gly-Pro

**Figure 2:**
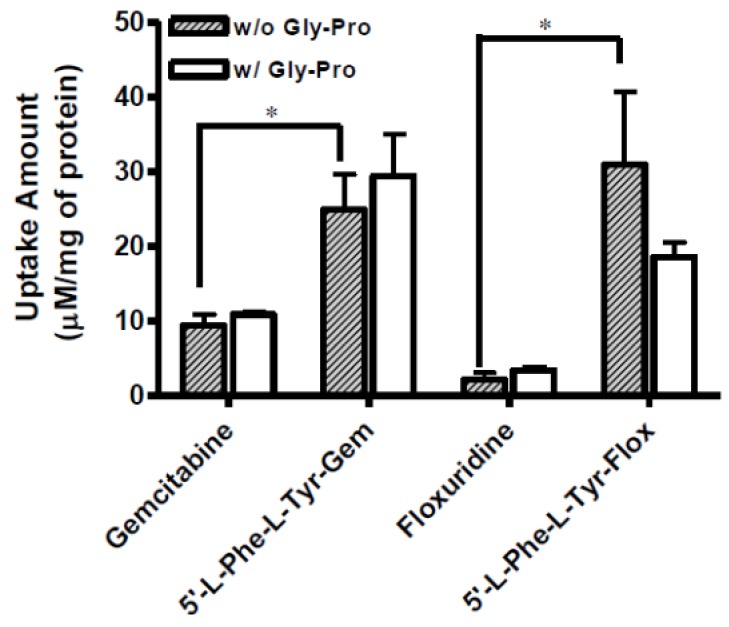
The uptake study with floxuridine, 5′-l-phenylalanyl-l-tyrosyl-floxuridine, gemcitabine, and 5′-l-phenylalanyl-ltyrosyl-gemcitabine found in Caco-2 cells with 1 h incubation with both the presence and the absence of 10 mM Gly-Pro. Each column represents total amount of floxuridine/gemcitabine prodrug, floxuridine/gemcitabine, and 5-FU/cytosine. Data are expressed as the amount, µM/mg of protein, mean ± SD, *n* = 3. * *p* < 0.05, the uptake amount of dipeptide prodrugs is compared with their parents, floxuridine and gemcitabine.

**Figure 3:**
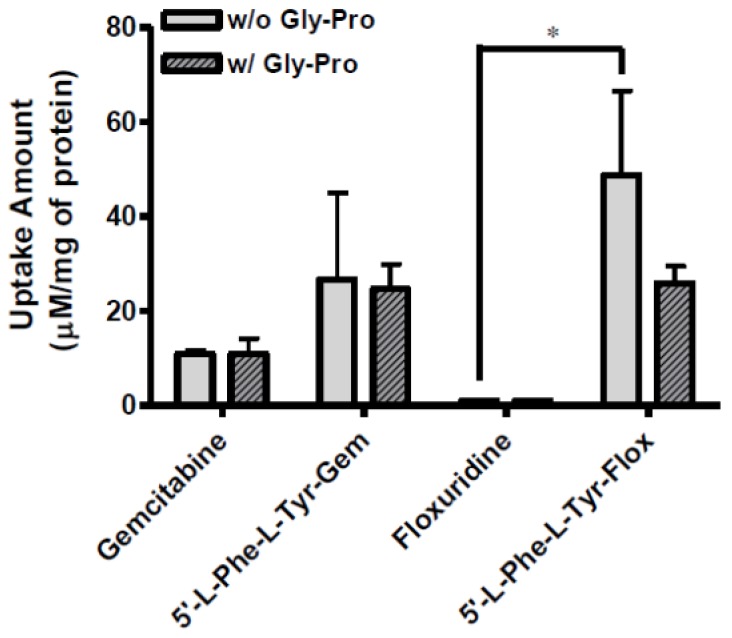
The uptake study with floxuridine, 5′-l-phenylalanyl-l-tyrosyl-floxuridine, gemcitabine, and 5′-l-phenylalanyl-l-tyrosyl-gemcitabine found in Panc-1 cells with 1 h incubation with the presence and the absence of 10 mM Gly-Pro. Each column represents the total amount of floxuridine/gemcitabine prodrug, floxuridine/gemcitabine, and 5-FU/cytosine. Data are expressed as the amount, µM/mg of protein, mean ± SD, *n* = 3. * *p* < 0.05, the uptake amount of dipeptide prodrugs is compared with their parents, floxuridine and gemcitabine.

**Figure 4:**
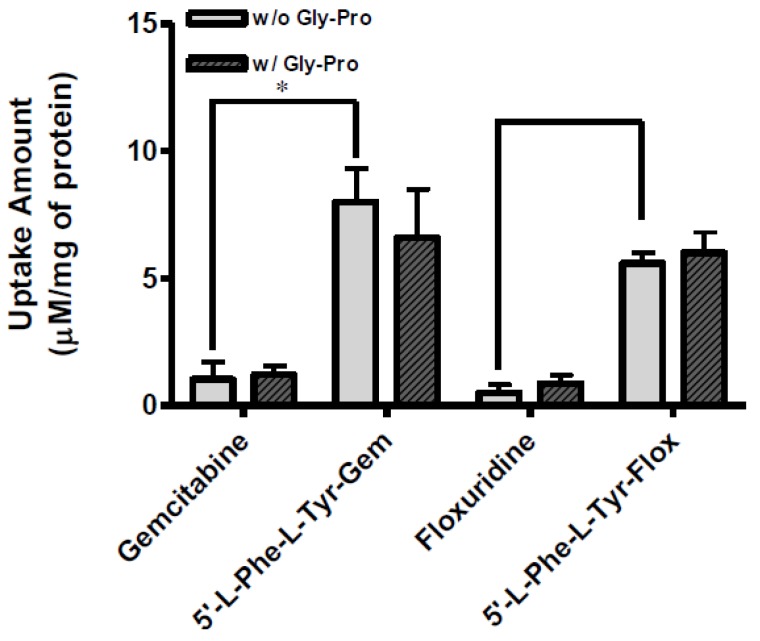
The uptake study with floxuridine, 5′-l-phenylalanyl-l-tyrosyl-floxuridine, gemcitabine, and 5′-l-phenylalanyl-l-tyrosyl-gemcitabine found in AsPC-1 cells with 1 h incubation with the presence and the absence of 10 mM Gly-Pro. Each column represents the total amount of gemcitabine prodrug, gemcitabine, and cytosine. Data are expressed as the amount, µM/mg of protein, mean ± SD, *n* = 3. * *p* < 0.05, the uptake amount of dipeptide prodrugs is compared with their parents, floxuridine and gemcitabine.

The uptake of dipeptide monoester prodrugs of floxuridine and gemcitabine and their parents, floxuridine and gemcitabine, was determined with both the presence and the absence of 10 mM Gly-Pro at 37 °C in Caco-2, Panc-1, and AsPC-1 cells. Figure 2 and Table 3 show the uptake amounts with observed compound species and the uptake difference with both the presence and the absence of 10 mM Gly-Pro in Caco-2 cell system. Figure 3 and Table 3 and Figure 4 and Table 3 show the uptake amounts with observed compound species in Panc-1 and AsPC-1, ductal pancreatic cancer cell lines, cell systems, respectively. Dipeptide prodrugs of floxuridine and gemcitabine exhibited 2.4- to 48.7-fold higher uptake amount than their parents, floxuridine and gemcitabine in Caco-2, Panc-1, and AsPC-1 cells. The uptake amount of floxuridine was not observed in Panc-1 cells, while the one of 5′-l-phenylalanyl-l-tyrosyl-floxuridine exhibited 48.7 µM/mg of protein in the same cell system. 5′-l-Phenylalanyl-l-tyrosyl-gemcitabine exhibited 2.4-fold better uptake in Panc-1 cell than their parent, gemcitabine (Figure 3). 5′-l-Phenylalanyl-l-tyrosyl-floxuridine and 5′-l-phenylalanyl-l-tyrosyl-gemcitabine exhibited 11.2- and 8.0-fold better uptakes than their parents in AsPC-1 cell, which reportedly overexpress PEPT1 transporter [35]. However, those uptake amounts of dipeptide prodrugs and parents did not show meaningful difference between the presence and the absence of Gly-Pro even though the dipeptide promoiety, 5′-l-phenylalanyl-l-tyrosine, exhibited the affinity to the PEPT1 transporter to inhibit glycylsarcosine (Gly-Sar) uptake [11]. Those results suggest that the improved cellular uptake of prodrugs in those cell lines was attributed not only to the carrier mediated processes but also to increased passive processes by prodrug design. The uptake amounts of dipeptide prodrugs were significantly lower in AsPC-1 cells compared to their corresponding values in Caco-2 and Panc-1 cells. Gemcitabine was degraded almost three- to four- times faster in AsPC-1 cells than in Caco-2 and Panc-1 cells, while floxuridine was degraded two- to six-times faster in AsPC-1 cells than in those cells. Those indicate that prodrug stability and enzyme upregulation in AsPC-1 cells would be largely attributed to the observed uptake amount for floxuridine and gemcitabine prodrugs and their metabolites. Those uptake results exhibited different pattern of drug species in those three cell lines suggesting the different rates for membrane permeability, enzymatic activation and metabolism. The uptake of dipeptide prodrugs in Caco-2 cell exhibited 6%–45% of prodrug form, 47%–91% of parent drug form and 3%–8% of metabolite, 5-FU and cytosine, while the uptake of dipeptide prodrugs in Panc-1 cell exhibited 9%–98% of prodrug form, 2%–15% of parent drug form and 0%–76% of metabolite (Table 3). In AsPC-1 cell, the uptake of dipeptide prodrugs in AsPC-1 cell exhibited 39%–60% of prodrug form, 0%–33% of parent drug form and 28%–40% of metabolite (Table 3). Those results suggested that dipeptide floxuridine prodrug is more stable than dipeptide gemcitabine prodrug even though the promoiety for those prodrugs is the same. However, floxuridine, its parent drug, was less stable than gemcitabine. When the floxuridine prodrug was metabolized to floxuridine, floxuridine was quickly metabolized to 5-FU by enzymes like thymidine phosphorylase and, therefore, only 0%–47% of floxuridine was observed compared to 15%–91% of gemcitabine in those uptake studies (Table 3). Those results suggest that the expression level of metabolizing enzymes in those cells for prodrugs and their parents was different. As a result, the bioactivation rates for those prodrugs and parents were different.

**Table 3:** Composition of floxuridine, floxuridine prodrug, gemcitabine and gemcitabine prodrug in Caco-2, Panc-1, and AsPC-1 cell uptake studies (mean ± SD, *n* = 3).

Compound	Cells	Metabolite * (%)	Parent drug * (%)	Prodrug (%)
Gemcitabine	**Caco**-**2**	5	95	-
5′-l-Phenylalanyl-l-tyrosyl-gemcitabine		3	91	6
Floxuridine		100	0	-
5′-l-Phenylalanyl-l-tyrosyl-floxuridine		8	47	45
Gemcitabine	**Panc**-**1**	80	20	-
5′-l-Phenylalanyl-l-tyrosyl-gemcitabine		76	15	9
Floxuridine		0	0	-
5′-l-Phenylalanyl-l-tyrosyl-floxuridine		0	2	98
Gemcitabine	**AsPC**-**1**	10	90	-
5′-l-Phenylalanyl-l-tyrosyl-gemcitabine		28	33	39
Floxuridine		67	33	-
5′-l-Phenylalanyl-l-tyrosyl-floxuridine		40	0	60

* Metabolite and parent drug are referred to cytosine and gemcitabine for a gemcitabine prodrug and to 5-FU and floxuridine for a floxuridine prodrug, respectively.

### 3.4. In Situ Permeability of Floxuridine, 5′-l-Phenylalanyl-l-Tyrosylfloxuridine, Gemcitabine, and 5′-l-Phenylalanyl-l-Tyrosylgemcitabine in the Single-Pass Intestinal Perfusion Study and the Drug Concentration in Plasma in Mice

**Figure 5:**
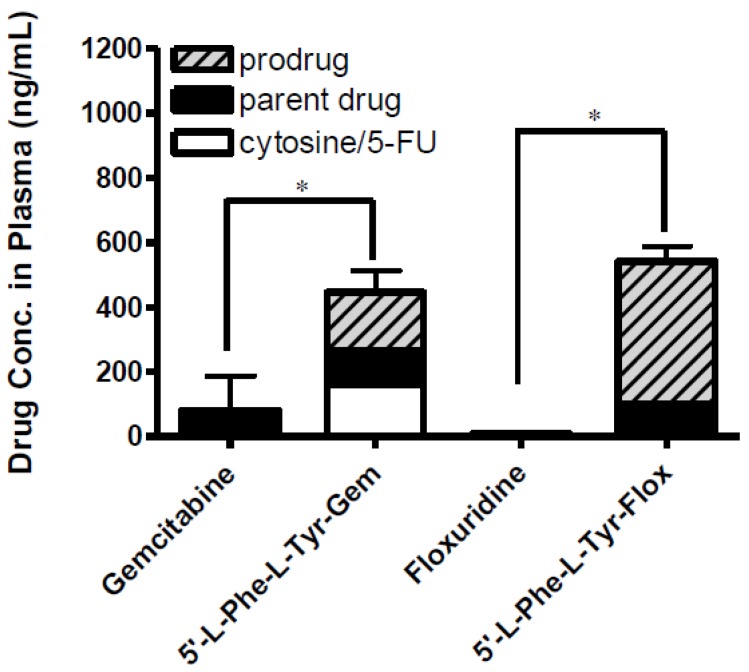
The concentration of floxuridine, floxuridine prodrug, gemcitabine and gemcitabine prodrug found in plasma at 2 h following *in situ* single-pass intestinal perfusion to the mouse jejunum. Each column represents prodrug, its parent drug, and its metabolite. Data are expressed as the concentration, ng/mL, mean ± SD, *n* = 3. Error bars are shown for the total concentration. * *p* < 0.05, the uptake amount of dipeptide prodrugs is compared with their parents, floxuridine and gemcitabine.

**Figure 6:**
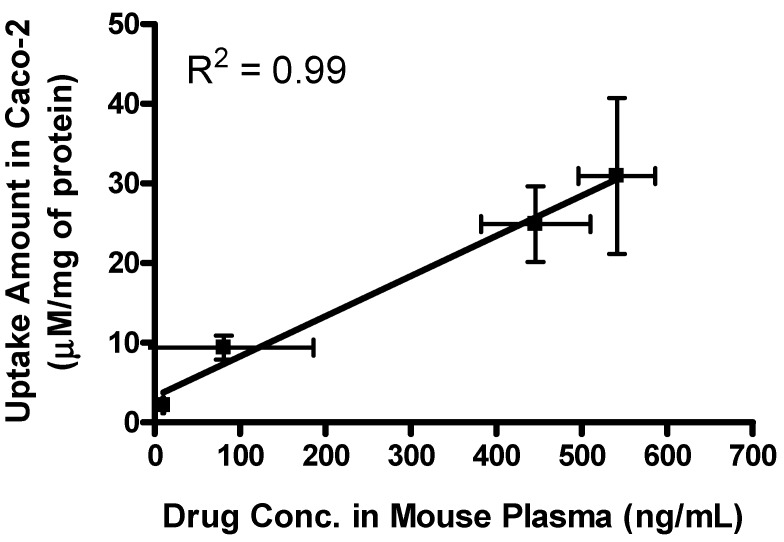
The correlation between the drug concentration in mouse plasma after *in situ* perfusion and the uptake amount of dipeptide prodrugs and their parents, floxuridine and gemcitabine, in Caco-2 cells. Values are the mean ± SD, *n* = 3.

The effective permeability (P_eff_) values obtained for floxuridine, 5′-l-phenylalanyl-l-tyrosyl-floxuridine, gemcitabine, and l-phenylalanyl-l-tyrosylgemcitabine in small intestinal segments in mice at physiological pH are presented in Table 4. The *in situ* permeability of parent drugs, floxuridine and gemcitabine, exhibited 0.1 × 10^−5^ cm/s and 0.2 × 10^−5^ cm/s in the mouse jejunum, respectively. On the other hand, the *in situ* permeability of dipeptide monoester prodrugs of floxuridine and gemcitabine exhibited 19.0- to 11.0-fold higher membrane permeability than their parent drugs (Table 4). The gemcitabine prodrug exhibited superior membrane permeability to the floxuridine prodrug. 5′-l-phenylalanyl-l-tyrosylfloxuridine displayed the highest drug concentration in mouse plasma after *in situ* perfusion and dipeptide prodrugs exhibited 53.0- and 5.5-fold higher drug concentration in mouse plasma than their parents, respectively. The majority (81%) of totally observed amount of a floxuridine prodrug in the systemic circulation was 5′-l-phenylalanyl-l-tyrosylfloxuridine after *in situ* perfusion study in mice, while only 40% of totally observed amount of a gemcitabine prodrug was 5′-l-phenylalanyl-l-tyrosylgemcitabine (Figure 5). Interestingly, 5-FU, the first metabolite of floxuridine, from 5′-l-phenylalanyl-l-tyrosylfloxuridine was not observed, while cytosine, the first metabolite of gemcitabine, from 5′-l-phenylalanyl-l-tyrosyl-gemcitabine was detected and exhibited one-third of totally observed amount. For parent drugs, floxuridine and gemcitabine, the majority (83%) of totally observed amount was gemcitabine, while floxuridine was only drug observed in mouse plasma after *in situ* perfusion. Those results suggested that the faster metabolism of floxuridine and 5-FU compared to gemcitabine and cytosine and agreed with *in vitro* stability studies in cell homogenates (Table 2). The effective permeability (P_eff_) values of dipeptide prodrugs as well as their parent drugs in mouse jejunal intestine are consistent with the trends observed in uptake studies in Caco-2 cells. The excellent correlation between the uptake amount in Caco-2 cells and the drug concentration in mouse plasma after *in situ* perfusion was observed even though sample points were limited (*R^2^* = 0.99), which agreed with our previous findings (Figure 6) [52].

**Table 4:** Effective (P_eff_) permeability coefficients of floxuridine, floxuridine prodrug, gemcitabine and gemcitabine prodrug in *in situ* perfusion study in mouse (mean ± SD, *n* = 3).

Prodrug/drug	Peff, mouse perfusion (×10^−5^ cm/s)
Gemcitabine	0.2 ± 0.2
5′-l-Phenylalanyl-l-tyrosyl-gemcitabine	2.2 ± 0.4 *
Floxuridine	0.1 ± 0.8
5′-l-Phenylalanyl-l-tyrosyl-floxuridine	1.9 ± 0.1 *

* *p* < 0.05, the permeability of dipeptide prodrugs is compared with their parents.

### 3.5. Cell Proliferation Assay

GI_50_ values for floxuridine, 5′-l-phenylalanyl-l-tyrosylfloxuridine, gemcitabine, and 5′-l-phenyl-alanyl-l-tyrosylgemcitabine, determined in cell proliferation studies with the pancreatic ductal cell lines, AsPC-1 and Panc-1, are shown in Table 5. Dipeptide prodrugs exhibited 2.0- to 2.7-fold enhanced anti-proliferative activity in AsPC-1 cell compared to their parents, floxuridine and gemcitabine. The parent drugs did not show any anti-proliferative activity in Panc-1 cell within the tested drug concentrations, while dipeptide prodrugs of those parent drugs exhibited around 3 mM of GI_50_ values (Table 5). The cell proliferation studies in the pancreatic ductal cancer cell lines confirmed the enhanced potency of the dipeptide monoester prodrugs compared to parents, floxuridine and gemcitabine. The results of uptake study in AsPC-1 and Panc-1 cells suggested that the enhanced anti-proliferative effect on cancer cells attributed the improved membrane permeability of a floxuridine prodrug and a gemcitabine prodrug regardless of carrier-mediated transporters. The GI_50_ values of prodrugs did not exhibit any discernible correlations with their uptake values in AsPC-1 and Panc-1 cells. Since the bioconversion rates of prodrugs and the metabolic rates of prodrugs and their parent drugs will be various, the time of transported drugs/prodrugs into cancer cells and of activated prodrugs to reach their maximum anti-proliferation activity will be different [47]. Therefore, to distinguish a significant correlation between GI_50_ values and prodrug/drug permeabilities with a limited experimental time course would be extremely difficult.

Despite this improvement of modified nucleoside analogs in membrane permeability and anti-proliferative activity, the delivery of those chemotherapeutic agents to the systemic circulation would not be enough to reach the same level of drug concentration obtained by intravenous administration, which steadily distributes higher drug concentration in the systemic circulation. However, this less exposure of chemotherapeutic agent might be the better way to treat cancers. Maximum tolerated dose (MTD) of chemotherapeutic agents has been clinically used to treat various cancer patients with multiple short therapeutic regimens, which generally require prolonged breaks from this type of therapies because of toxic side effects. Instead of the treatment with MTD of chemotherapeutic agents, the treatments with low and more frequent doses of chemotherapeutic agents called metronomic chemotherapy have been praised for its better antitumor effects and less toxicity [53,54,55,56,57,58]. With this metronomic approach, the development of orally administrable cancer drugs might be more feasible and beneficial for cancer patients. Indeed, the pre-clinical and clinical studies including metronomic therapy with other gemcitabine derivatives such as SL-01, CP-4126 and LY2334737, which are orally administrable gemcitabine prodrugs, have been conducted and have demonstrated therapeutic benefits of those prodrugs over their parent, gemcitabine [59,60,61,62,63,64,65,66]. CP-4126 and 5′-l-phenylanalyl-l-tyrosyl-gemcitabine contain a fatty acid chain and a dipeptide at their 5′ position, respectively, while nothing is bound to LY2334737 and SL-01 at their 5′ position. Most of chemotherapeutic agents like gemcitabine are DNA-damaging agents to inhibit or even kill rapidly dividing cells. Gemcitabine is generally administered intravenously in the clinic weekly basis and incorporated into DNA to apoptosis [64,67,68,69,70]. Gemcitabine and its prodrugs, SL-01 and LY2334737, can be phosphorylated and incorporated into DNA without any molecular modification because of free 5′ position. On the other hand, the promoiety of gemcitabine prodrugs, dipeptide prodrugs and CP-4126, at 5′ position has to be cleaved and phosphorylated before incorporated into DNA. This metabolic process could be an advantage to improve the tumor selectivity and targetability when, especially, tumor-related enzymes in tumor cells specifically metabolize a prodrug to release its parent drug. Indeed, prodrugs have been designed to be activated by enzymes that are specifically expressed in tumor in order to improve the tumor selectivity and, hence, to minimize the toxic effect at non-tumor sites [46,71,72,73,74,75,76,77]. The dipeptide prodrugs of floxuridine, 5′-l-phenylanalyl-l-tyrosyl-floxuridine, as well as gemcitabine, 5′-l-phenylanalyl-l-tyrosylgemcitabine, might be favorably activated by the enzyme which is highly up-regulated in the tumor cells [47].

Nucleoside transporters are believed to be responsible transporting nucleoside analogs like gemcitabine into cancer cells [78,79]. Delivery of gemcitabine into tumor cells relies on intake transporters such as nucleoside transporters (ENTs). However, the cancer treatment with nucleoside analogs like gemcitabine leads to down-regulation of ENTs and up-regulation of efflux transporters, multidrug resistance proteins (MRPs), in tumor cells and, as a result, those tumors exhibit the chemoresistance against chemotherapeutic medications [80,81,82,83]. In some cancers, up-regulation or high expression of nutrient transporters has been reported but down-regulation of those has not been reported [35,84,85,86]. Therefore, amino acid and dipeptide prodrugs of chemotherapeutic agents might have another advantage transporting drugs into cancer cells via influx transporters such as LATs, and PEPTs except ENTs [7,11,16,87]. It has been reported that the chemoresistance is attributed to the up-regulation of MRPs, especially MRP5 [80,81,88,89,90]. Numerous studies have been conducted to overcome this resistance to treat cancers but clinically successful approaches have not been established [91,92,93,94,95]. Systemic chemotherapy has significantly improved the therapeutic index in cancers but the development of drug resistance limits the chemotherapeutic efficacy and further improvement. The drug resistance in chemotherapy is developed in very complex process and the mechanism of this chemotherapeutic resistance should be studies and understood for better cancer treatment.

**Table 5:** Cell Growth Inhibition in AsPC-1 and Panc-1 Cells by floxuridine, floxuridine prodrug, gemcitabine, and gemcitabine prodrug (mean ± SD, *n* = 3–5).

Prodrug/drug	GI_50_ AsPC-1 (mM)	GI_50_ Panc-1 (mM)
Gemcitabine	10.2 ± 1.6	ND
5′-l-Phenylalanyl-l-tyrosyl-gemcitabine	5.0 ± 0.3	3.2 ± 0.7
Floxuridine	22.9 ± 5.7 ^#^	ND
5′-l-Phenylalanyl-l-tyrosyl-floxuridine	4.2 ± 0.1	3.0 ± 0.3

ND-no significant inhibitory effect was observed. ^#^ Ref. [11].

## 4. Conclusions

The observed species of floxuridine prodrugs and gemcitabine prodrugs in cellular uptake studies may illustrate that transported drugs are converted to floxuridine and 5-FU and to gemcitabine and cytosine via a sequential enzymatic pathway, respectively [11,50]. The dipeptide prodrugs of nucleoside analogs, floxuridine and gemcitabine, demonstrated the superior membrane permeability to their parent drugs. As a result, those prodrugs exhibited higher concentration of cancer drugs in pancreatic cancer cells, AsPC-1 and Panc-1, and better anti-proliferative activity. Our results indicate that dipeptide monoester prodrugs of floxuridine and gemcitabine exhibit significantly higher permeability in mouse intestinal membrane than their parent drugs, floxuridine and gemcitabine, which agreed with our previous findings in the membrane permeability across Caco-2 monolayers [11]. Also, uptake amounts of those prodrugs exhibited more in three different cells than ones of their parent drugs. Dipeptide gemcitabine prodrug, 5′-l-phenylanalyl-l-tyrosylgemcitabine, displayed significantly higher permeability in mouse intestinal membrane but the other dipeptide prodrug, 5′-l-phenylanalyl-l-tyrosyl-floxuridine exhibited the highest prodrug concentration in blood with slightly better anti-proliferative activity against pancreatic ductal cancer cells, Panc1 and AsPC-1. Both prodrugs clearly exhibited superior membrane permeability and, hence, anti-proliferative activities to their parents. With the difference in enzymatic stability between prodrug and parent drug, the 5′-l-phenylanalyl-l-tyrosylfloxuridine has an advantage in prodrug delivery into the systemic circulation over 5′-l-phenylanalyl-l-tyrosylgemcitabine. However, its metabolite, floxuridine, is quickly metabolized further in the systemic circulation. On the other hand, gemcitabine displayed more enzymatic stability in the systemic circulation and might have more advantage over floxuridine as an orally administrable chemotherapeutic agent. This question might be addressed by pharmacokinetics studies. Taken together, the dipeptide prodrugs of nucleoside analogs exhibited enhanced membrane permeability, the higher prodrug concentration in plasma after *in situ* perfusion, and the improved *in vitro* anti-cancer effect. The improvements would prove the feasibility for the development of oral dosage form for pyrimidine analogues. However, the stability and permeability of those prodrugs have to be improved more in order to achieve the therapeutic drug concentration to inhibit tumor growth *in vivo*. Current data exhibits that oral prodrug dosage forms cannot simply replace the intravenous administration of chemotherapeutic agents. With careful investigation of dose optimization, dosage regimen, and combination therapy, those prodrugs might be more suitable for metronomic chemotherapy than one for orthodox chemotherapy. Prodrug approaches provide a powerful tool to improve physicochemical properties of drug with chemical modification and conjugation for better therapeutic efficacy. The cancer drugs with improved oral bioavailability should easily improve the patient compliance and also improve the life flexibility for cancer patients, who often have to visit the hospital for their chemotherapeutic regiments because of intravenous treatment.

## Supplementary Files

Supplementary File 1 Supplementary Materials (DOCX, 293 KB)
