# Supplementary material for: The Dipeptide Monoester Prodrugs of Floxuridine and Gemcitabine—Feasibility of Orally Administrable Nucleoside Analogs
Source: Pharmaceuticals (Basel). 2014 Jan 27;7(2):169–91. doi: 10.3390/ph7020169 (PMC3942691; doi:10.3390/ph7020169)
Supplement: Supplementary File 1 — Supplementary Materials (DOCX, 293 KB) [file pharmaceuticals-07-00169-s001.docx]

**Supplementary Materials**

**Figure S1.** HPLC Chromatograph of 5′-l-phenylalanyl-l-tyrosyl-gemcitabine.


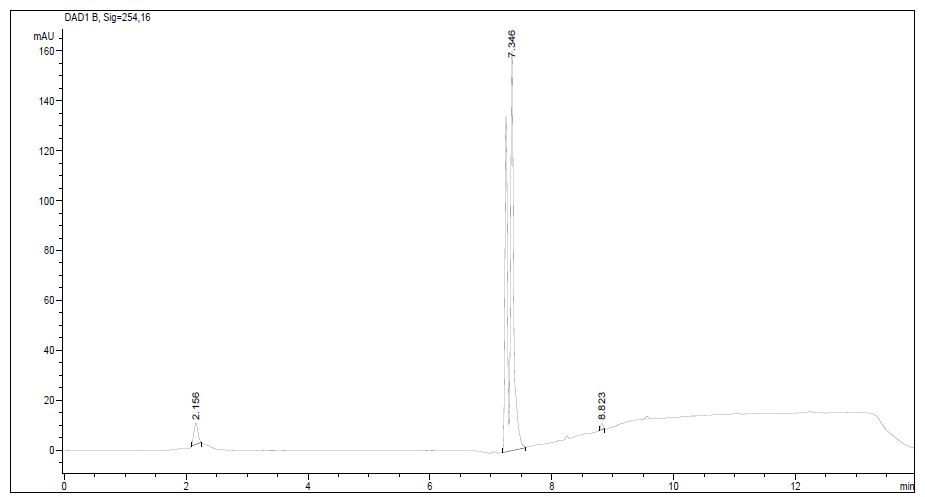


| **Time (min)** | **Area (%)** | **Area (%)** | **Compound** |
| --- | --- | --- | --- |
| 2.156 | 39.6 | 3.9 | Gemcitabine |
| 7.346 | 946.6 | 95.5 | 5′-Phe-Tyr-Gem |
| 8.823 | 6.2 | 0.6 | - |

**Figure S2.** ^13^C-NMR spectrum of 5′-l-phenylalanyl-l-tyrosyl-gemcitabine.


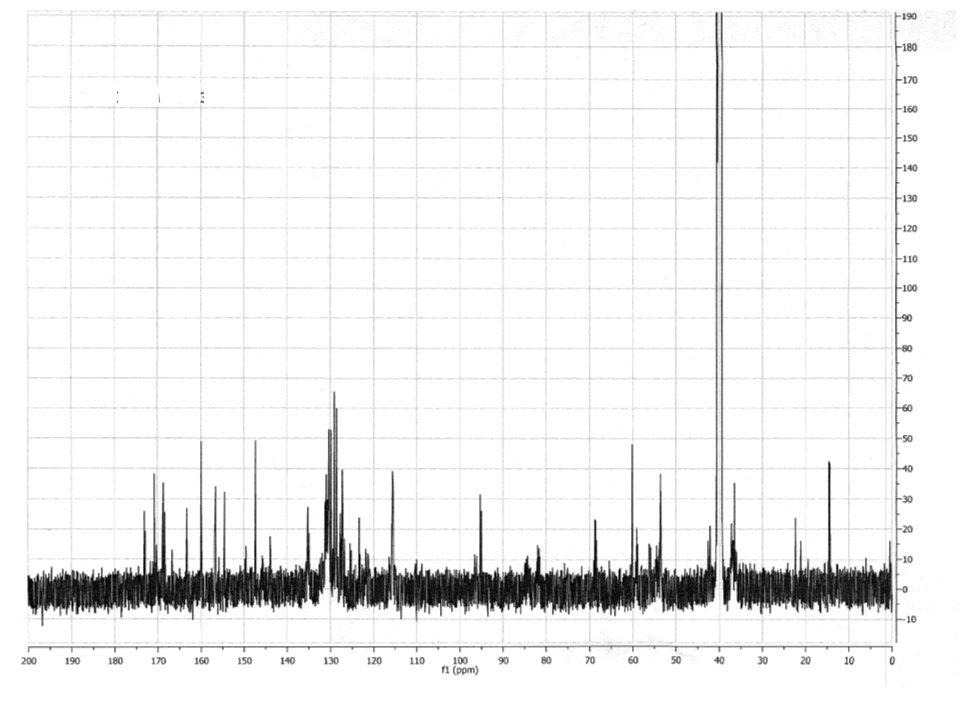


Mixture of Diastereomers

^13^C-NMR (126 MHz, DMSO) δ 173.09, 172.89, 170.77, 170.24, 168.71, 168.36, 166.71, 166.60, 163.20, 159.93, 156.69, 156.59, 154.54, 154.49, 147.29, 143.90, 135.17, 131.18, 130.95, 130.34, 129.86, 129.09, 128.59, 127.76, 127.26, 126.84, 125.46, 123.41, 121.34, 115.56, 115.39, 95.30, 95.07, 68.87, 68.67, 68.50, 60.20, 59.15, 56.34, 53.63, 53.47, 22.40, 14.63, 14.45.
